# "Seeing a doctor is just like having a date": a qualitative study on doctor shopping among overactive bladder patients in Hong Kong

**DOI:** 10.1186/1471-2296-15-27

**Published:** 2014-02-06

**Authors:** Judy Yuen-man Siu

**Affiliations:** 1 David C. Lam Institute for East–West Studies (Environment, Health, and Sustainability working group), Hong Kong Baptist University, Kowloon Tong, KLN, Hong Kong

**Keywords:** Hong Kong, Doctor shopping, Perceptions, Social environment, Illness and treatment experiences, Personal cultural preference, Cultural beliefs, Overactive bladder patients

## Abstract

**Background:**

Although having a regular primary care provider is noted to be beneficial to health, doctor shopping has been documented as a common treatment seeking behavior among chronically ill patients in different countries. However, little research has been conducted into the reasons behind doctor shopping behavior among patients with overactive bladder, and even less into how this behavior relates to these patients’ illness and social experiences, perceptions, and cultural practices. Therefore, this study examines overactive bladder patients to investigate the reasons behind doctor shopping behavior.

**Methods:**

My study takes a qualitative approach, conducting 30 semi-structured individual interviews, with 30 overactive bladder patients in Hong Kong.

**Results:**

My study found six primary themes that influenced doctor shopping behavior: lack of perceived need, convenience, work-provided medical insurance, unpleasant experiences with doctors, searching for a match doctor, and switching between biomedicine and traditional Chinese medicine. Besides the perceptual factors, participants’ social environment, illness experiences, personal cultural preference, and cultural beliefs also intertwined to generate their doctor shopping behavior. Due to the low perceived need for a regular personal primary care physician, environmental factors such as time, locational convenience, and work-provided medical insurance became decisive in doctor shopping behavior. Patients’ unpleasant illness experiences, stemming from a lack of understanding among many primary care doctors about overactive bladder, contributed to participants’ sense of mismatch with these doctors, which induced them to shop for another doctor.

**Conclusions:**

Overactive bladder is a chronic bladder condition with very limited treatment outcome. Although patients with overactive bladder often require specialty urology treatment, it is usually beneficial for the patients to receive continuous, coordinated, comprehensive, and patient-centered support from their primary care providers. Primary care doctors’ understanding on patients with overactive bladder with empathetic attitudes is important to reduce the motivations of doctor shopping behavior among these patients.

## Background

The phenomenon of "doctor shopping" has been documented in many countries, and is a common treatment seeking behavior among patients. While usage varies within previous literature, in general, the term can refer to the following: (a) changing doctors without professional advice or referral during a single illness episode [1]; (b) consulting two or more doctors for a single disease [2,3]; (c) visiting a clinic with multiple doctors for the same disease [4]; and (d) using several doctors simultaneously [5]. No matter which definition, doctor shopping can result in the wastage and depletion of health resources [6,7].

Having a regular primary care provider, or a family doctor, has been found to be beneficial to overall health [6]. Primary care doctors offer "10 Cs" of patient care: continuity, comprehensiveness, coordination, first-contact, competence, cost-effectiveness, communication, collaboration, compliance, and competing demands [8]. This is particularly important for people with chronic conditions because they often require long-term continuity of care [9]. However, literature shows that doctor shopping is common among chronically ill patients [10], especially in non-western countries where the family doctor tradition is less established [9,11]. Doctor shoppers also tend to be younger and better informed about medical specialties [12]. Previous literature points to persistence of symptoms, distrust about medications, seeking a second opinion, and family’s and friends’ advice as the most common reasons for doctor shopping [6]. Such behavior reflects that the needs of patients are not being sufficiently addressed [13].

Overactive bladder (OAB) is a common chronic bladder dysfunction in Hong Kong. Although OAB and its symptoms are not well-defined [14], urinary frequency, urgency, and incontinence are considered as the most typical symptoms [15]. Treatment and management plan for OAB can include lifestyle modifications, behavioral therapy, pharmacotherapy, neuromodulation, botulinum toxin therapy, and surgical interventions [16]. It is estimated that around 15% of the Hong Kong population suffer from OAB [17]. Due to the high prevalence, primary care doctors are likely to encounter OAB patients [18]. OAB often requires long-term treatment, and it can seriously affect patients’ physical, psychological, social, and sexual wellbeing [19,20]. Because of its widespread effects, it is suggested to be best managed by regular healthcare providers who can offer continuous care and are familiar with available treatment options, leading to accurate diagnosis and effective treatment [18]. Adopting a patient-centered approach to symptom management is also recommended to optimize treatment outcomes and quality of life [21].

However, unpleasant experiences prevail for OAB patients who seek medical treatment for their bladder condition. Miscommunication [22,23] and misunderstandings [24] between patients and treatment providers are common, and patients are often dissatisfied with the care they receive [23]. These factors are shown to greatly influence patients’ doctor shopping behavior [2].

### Significance

Although OAB is a common chronic bladder dysfunction, research about OAB patients’ life is lacking. Doctor shopping behavior among OAB patients is an understudied area. Little research has been conducted into the reasons behind their doctor shopping, and even less into how this behavior relates to patients’ perceptions, illness and social experiences, personal cultural preference, and cultural practices. However, it is an important topic in both chronically ill primary care and OAB patient care, because of the negative impacts this behavior can have on their treatment compliance and outcome [25], as well as patient safety. In response to this literature gap and its significant implication on chronic patient care, this qualitative study was conducted to examine the reasons behind doctor shopping behavior by studying the example of OAB patients in Hong Kong.

My attention was first drawn to this area of study by my cousin, a longtime sufferer of OAB. She saw many different primary care doctors for her condition; however, these were frequently difficult and embarrassing experiences, and she even faced blame from some of her doctors. She eventually visited a private-practice urologist without referral and was finally diagnosed with OAB, seven years after the onset of symptoms.

My cousin encountered many painful experiences throughout her treatment-seeking journey in primary care. Her story inspired me to find out if other patients with OAB had undergone similar experiences. I also wondered whether primary care doctors in Hong Kong had a good understanding and awareness of OAB, since primary care doctors are supposed to be the first stop of treatment seeking. I therefore became interested in investigating the illness experiences of this "minority" group of chronically ill patients, who were not well recognized and received very little attention or support from their society. With better understanding of the doctor shopping behavior of OAB patients in Hong Kong their needs can be better addressed, enabling the provision of better care and support to these patients, in order to ultimately reduce their incentive to doctor shop and improve their treatment experiences.

## Methods

The study takes a qualitative approach, collecting data using in-depth individual semi-structured interviews between May and August 2012. Thirty people diagnosed with OAB were recruited from an OAB patient self-help group in Hong Kong by purposive sampling.

### Ethical considerations

I obtained ethics approval from the Committee on the Use of Human and Animal Subjects in Teaching and Research of the Hong Kong Baptist University prior to the study. Participation in the study was purely voluntary. Information sheets explaining the purpose and nature of the study were provided to all participants, written in the participants’ mother tongue – traditional Chinese – to ensure clear understanding. I also provided verbal explanation and clarification prior to the interviews, and obtained written consent from each of the participants. They were assured of their rights and freedom to withdraw from the study without prejudice. No identifying details were recorded in the audio records or the coded data to ensure participant privacy, and all interview transcripts were marked with codes. The data was stored in locked files and treated with strict confidentiality. The audio records of the interviews were destroyed after the interviews had been transcribed.

### Participants

The 30 participants were all members of an OAB patient self-help group in Hong Kong, and included 19 females and 11 males between the ages of 32 and 58. They were employed in civil service, administration and executive functions, commerce, education, social service, information technology, and the service industry. All the participants were chosen using purposive sampling, and fit the following criteria: (a) were diagnosed with OAB by a medical practitioner; (b) had been to primary care doctors for their bladder symptoms; (c) had not had a regular primary care doctor in the past five years; and (d) were ethnically Hong Kong Chinese. Those diagnosed with other types of urinary incontinence (including stress incontinence, overflow incontinence, mixed incontinence, structural incontinence, and functional incontinence), and those without a confirmed diagnosis of OAB prior to the sampling period were excluded from this study.

The length of time since diagnosis ranged from 1 to 6 years at the time of the study, though participants had been suffering from the symptoms of urinary frequency, urgency, and incontinence between 5 and 11 years. None of the participants received a correct diagnosis immediately after their symptoms emerged, and all suffered symptoms for at least two years, and up to five, before they were diagnosed with OAB. In those intervening years, all participants performed primary care doctor shopping for their bladder condition.

After they received a confirmed diagnosis of OAB, participants required long-term follow-up treatment in the urology specialty clinics of public hospitals. Yet primary care doctor shopping remained common even after diagnosis, in seeking treatment for their bladder conditions and also other ailments. The participants shopped for different doctors in an attempt to find a physician who could understand their bladder symptoms, even when seeking treatment for other conditions.

### Data collection

I conducted all the interviews in Cantonese Chinese, which is my own mother tongue and that of the participants. This ensured that participants could express their views and experiences freely without language barriers. I conducted all the interviews myself, which guaranteed the consistency and quality of each interview. This avoided interview inconsistency, and data flaw and inadequacy by appointing outsider person as the interviewer. The interviews were open-ended in nature, and the participants had a high degree of flexibility to express their views, feelings and experiences [26].

The interviews were conducted on an individual basis in a private room of the patient self-help group, between May and August 2012. Prior to the interviews, I developed an interview question guide to ensure the interviews were on focused topics and followed an appropriate direction. The guide contained a set of open-ended questions investigating the reasons behind participants’ doctor shopping behavior, and their experiences seeking treatment in primary care:

1. What are the reasons you do not have a regular primary care doctor?

2. What was your experience (positive and negative) when you were seeking primary care treatment?

3. Who did you consult first for your bladder condition? Why? How was your experience?

4. How many primary care doctors have you been to for your bladder complaint? How long have you sought treatment from primary care doctors for your bladder condition?

5. What did the primary care doctors do about your bladder condition? How did you feel about what they did?

6. Why did you switch to another doctor for your bladder condition?

7. What was your experience (positive and negative) with these primary care doctors in dealing with your bladder condition?

8. How did you feel during your journey of seeking treatment before receiving specialist care?

9. Are there any differences in your feelings when you were seeking primary care and after receiving specialist care for your bladder condition?

10. Who diagnosed your OAB? How was your experience before you had been diagnosed with OAB? How was your experience after?

11. Have you ever sought types of treatment other than biomedicine for your bladder condition? How was that experience?

12. Does suffering from OAB change your habit of seeing doctors? If yes, how?

Additional follow-up questions were asked in response to different participant answers, to obtain more in-depth data. Each interview lasted between 1.75 and 2 hours, and was audio-recorded with consent. Because of their bladder conditions, the interviews were paused at the participants’ request. To compensate them for their time, each participant was given a supermarket cash coupon of HK$100 upon completion of the interview.

### Data analysis

Quick data analysis was made during the interviews to determine what was known and what needed to be explored further [27]. All interviews were transcribed verbatim and then translated into English. Thematic content analysis was performed [28]. Interview transcriptions were segmented into meaning units, collapsed and classified into categories subsequently and eventually themes through the process of abstraction and constant comparison. Categorical themes were classified and named as they emerged. Repetitive codes and themes were noted and highlighted. A coding scheme was developed [28] according to an inductive coding process by allowing the discovery of patterns of behaviors and thoughts [26]. New thematic codes were added to the coding list. Ideas, observational notes, commentary, and special data during the interviews were documented in a codebook [26] to assure cross-reference with the interview transcription as well as the consistency and accuracy of the data collected. A coding table which identified themes, categories, and codes with supporting interview quotes was constructed. Because I conducted all of the data collection and analysis in this study, a recoding process was performed one month after the first coding as cross-analysis in order to eliminate possible subjectivity and bias, and to enhance the validity and reliability of the coded data.

### Rigor

Data saturation was achieved. Validity checking was performed by asking the participants to read over their transcribed interviews to ensure accuracy and to achieve an emic understanding [27]. Reliability and confirmability were established through coding and recoding the transcripts to ensure that codings and categories were free of ambiguity, overlaps, and lack of clarity. Neutrality was achieved, with findings grounded in the interview data and not in any bias, motivation, or interest. Direct interview quotations were referenced in the analysis to clearly represent the ideas.

## Results

For the purposes of this study, doctor shopping behavior of the participants fits the following definitions: (a) changing doctors without professional advice or referral during a single illness episode [1]; (b) consulting two or more doctors for a single disease [2,3]; and (c) using several doctors simultaneously [5]. The subsequent six factors in relation to participants’ perceptions, social environment, illness experiences, personal cultural preference, and cultural beliefs resulted in their doctor shopping behavior.

### Perceptions

#### Lack of perceived needs

None of the participants perceived themselves to need a regular primary care doctor, because aside from their OAB, they believed their health to be generally good. They chose to receive long-term follow-up treatment for their OAB from public hospitals, and instead only consulted primary care physicians for illnesses they perceived to be "simple", such as the common cold, flu, and gastroenteritis. Doctor shopping was common in dealing with these "simple" ailments:

The biggest problem for me is my [overactive] bladder, but I am seeing specialty [clinic] in public hospitals already, so I do not need to go outside [to private practice doctors] for my bladder problem. My health is not bad though, just occasionally catch cold, flu, cough, or gastroenteritis. These diseases are very simple, and every doctor is able to treat these small problems, so I will just randomly go to any doctor for these small diseases without much thinking and choosing. [P2]

Most participants thought that they only required a regular doctor for more severe problems; so although they did not have a regular primary care doctor, most of them had a regular specialist. They did not have much motivation to switch specialists if they found one who could treat them, but they often performed doctor shopping in primary care when having "simple" diseases. This participant explained:

For the more severe problems I will see a regular specialist. Because the diseases are more serious, I have to find a good doctor and stick to him. If that doctor can treat my problems, then I will not want to switch. This is different for those simple diseases like cold, flu, and cough; I can just see a "street doctor" [general practitioner]. These are simple diseases and you do not need to care much. These simple diseases will not kill you, and every doctor is able to treat them. I will not go to these street doctors to treat my more serious problems, because I do not think they have the ability to treat these serious diseases – see how many street doctors I had been to for my bladder. However, I will not care much if I just have a cold or cough; every "street doctor" can do. It is not really a matter which doctor I see for simple diseases. [P3]

### Social environment

#### Convenience

Convenience was another significant factor contributing to participants’ doctor shopping. Because all the participants were employed, they mostly selected doctors located near their workplace for treatment, and shopped for a new doctor when they moved to a new job. The location of their homes was also important for them in selecting a doctor. As this participant mentioned:

The location [of a doctor’s clinic] is very important for me. If you are just having small diseases, then every doctor will be okay. Therefore when I get sick in the office, I will just randomly choose a doctor near my workplace. If I get sick at home, I will just randomly go to a doctor near my home. I will see another new doctor when I change to a new job or move to a new home, because the new workplace and new home will be different in most cases. You will not want to travel back to the original doctor for small diseases. [P12]

In addition to suitable and convenient locations, doctors’ consultation times were another crucial element in participants’ doctor shopping behavior. Consultation times that fit around participants’ work schedules were therefore decisive in selecting a doctor. This participant indicated how long working hours forced her to doctor shop:

It is not always easy to leave office to see a doctor during office hours. Very often I need to work overtime till late at night, and most doctors have closed already. Even if I want to see my own doctor, it is often impossible. I can only choose those doctors who are still open very late at night, such as those doctors working in chain clinics [health maintenance organizations], the 24-hour clinics in private hospitals, or the emergency room [accident and emergency department] of public hospitals. You will see different doctors each time when you go to these places for treatment. [P21]

#### Work-provided medical insurance

For those participants whose employers offered outpatient medical benefits, work-provided medical insurance was also a significant factor for them to doctor shop. In most cases, such medical insurance schemes offered a list of designated doctors who would provide medical care at a discounted rate. Some participants were therefore forced to switch doctors in order to make use of their work-provided medical insurance:

I used to see a regular doctor some years ago. However, after I began working at this company, I had to switch to the panel doctors [doctors designated by the work-provided medical insurance] so that I can enjoy the staff medical benefits. The panel doctors are not good. I tried many panel doctors but I failed to get treated. Therefore each time I get sick, I have to try a new panel doctor. Of course I can go back to my own doctor, but I will not be able to enjoy the medical benefit because he is not on the panel list [P16].

Other participants had negative treatment experiences with panel doctors that forced them to doctor shop. This participant reported:

My company joined the medical card scheme so I use medical card to see the panel doctors. I only need to pay a little by using medical card. Although the fee is really low, the service is terrible. The panel doctors do not give me any physical examination, and I can only stay in the doctor’s room for a minute. I am asked to leave once I have finished telling them about my problems. The worst thing is that the medication is totally ineffective. Very often I get even worse after seeing these doctors. Most panel doctors of my company’s medical card scheme are terrible, so I keep switching and switching. [P20]

### Illness experiences

#### Unpleasant experiences with doctors

Participants’ unpleasant experiences with primary care doctors served as a significant barrier in establishing long-term therapeutic relationships. More than half of the participants encountered unfriendly attitudes from primary care doctors in relation to their chronic bladder conditions, and these unpleasant experiences motivated them to keep shopping for a doctor who could understand their suffering. This participant shared how he had been misunderstood by different primary care doctors during his treatment journey:

I have been seeing many doctors for my bladder. However, none of them understood my problem. Some doctors wondered if I had been to "yellow places" [places offering sexual services], and said I’d better be honest with them. Some doctors kept focusing on if I have caught sex diseases [sexually-transmitted diseases]. Some doctors thought that I had emotional problems. Some doctors thought that I was making trouble for them, and they just asked me to go to another doctor without referral. I keep searching and trying different doctors, but none of them want to treat me. [P29]

Some participants also experienced teasing from primary care doctors when they were seeking treatment for their bladder condition, which drove them to doctor shopping. As this participant shared:

Some doctors teased me if I have taken "little K" [ketamine], while other doctors teased me about having "weak kidneys" [Cantonese slang referring to weak male sexual ability]. I felt very bad, and I felt insulted and disrespected. They are doctors, and I just cannot accept that they can tease patients in such a humiliating manner. I will just go to another doctor if they tease me. Unfortunately many doctors are not serious in my problem, so I need to keep switching doctors. [P8]

Apart from facing these unkind attitudes by their primary care providers, the participants’ treatment experiences were always unpleasant because of the long, arduous, and inefficacious treatment plans. This was often due to the doctors’ lack of knowledge regarding OAB, making them to do doctor shopping:

I have been trying many doctors, but none of them can treat me. They just did some check-ups and tested my urine. They would conclude that I just have bad voiding habit if they found nothing wrong from the [urinalysis] report. Some doctors would say I may have caught some very rare infection that may not be able to get tested so they gave me some antibiotics. Every time when I went to a new doctor, the doctor would do the same thing and said the same thing. The medication was useless, and no referral was made. I have to try different doctors by myself, but I just could not get treated. [P24]

### Personal cultural preference

#### Searching for a "match" doctor

The participants put a lot of emphasis on the concept of "match" in choosing a doctor. Such personal cultural preference was prevalent for almost all the participants and contributed to their doctor shopping behavior. One of the most important perceived elements of a good match was treatment efficacy. The participants’ failure in finding a doctor who could provide them efficacious treatment precipitated shopping to find a better match. This participant explained her perception of the term "match":

If a doctor can treat my problems quickly, then that means the doctor matches me. It is very important as I think everyone wants to recover quickly. After all, you need to pay to see a doctor. However, it is really difficult for me to find a match doctor even for very simple diseases like cold and flu, not even mentioning my bladder problems. Of course I will not go back [if the doctor fails to treat me]. Up till now I am still searching for a doctor who can match me. [P18]

Doctors’ attitudes as well as communication style were also commonly mentioned when the participants defined a match doctor. This participant used dating as an example for finding a good match:

Seeing a doctor is just like having a date. You will need to be able to communicate with the doctor to see if his personality and character match with yours. The doctor will be able to understand my needs and I will be able to understand his treatment if we can match each other. If we cannot match, how can we communicate? Also, doctor’s personality and character are important too. We will not be able to communicate if his attitude and temper are bad. However, not many doctors are willing to talk to patients. They just want to finish with a patient as quickly as possible. Some doctors even lose their temper if you ask more questions, and our relationship will be broken. I find it very difficult to find a doctor who can match me, so I am still searching [P13].

Although the participants no longer went to primary care doctors primarily for the treatment of OAB after they had been diagnosed, they emphasized the empathy of these doctors in understanding their bladder conditions. Failure to understand their bladder condition was considered a mismatch, and this motivated them to shop for another doctor:

Every time I see a new doctor, the doctor will ask me about my health history. Sometimes they could not understand what overactive bladder is, sometimes they would be shocked, and sometimes they would ask me some embarrassing questions such as if I have taken "little K" [ketamine] before. I always think seeing a doctor is not much different from speed dating, and you will know if the doctor can match with you in several minutes. If the doctor asks me some inconsiderate questions such as if I have taken "little K" before, then that means the doctor cannot understand me. Then how can we communicate, build up trust, and establish a long-term relationship with each other? [P29]

### Cultural beliefs

#### Switching between biomedicine and traditional Chinese medicine

In addition to seeking treatment from doctors in biomedicine, half of the participants also sought treatment from practitioners of traditional Chinese medicine (TCM). This choice contributed to doctor shopping behavior. In most cases, participants turned to TCM once they were close to finishing their biomedical treatment for the same disease. This participant shared her reasoning:

Western medicine [biomedicine] hurts the body. Therefore I go to a Chinese medicine practitioner to clear the "tail" [end of disease] and to rebalance the body. Sometimes I go to several western doctors to have treatment. In this case it makes my body weaker and more depleted as I have taken so many different types of western medications from different doctors. Also, I have to take western medications continuously for my bladder. Therefore I will go to a Chinese medicine practitioner for body rebalance as well. [P17]

Some participants received concurrent treatment from biomedical doctors and TCM practitioners for the same disease. The concurrent treatments caused them to shop for both biomedical doctors and TCM practitioners. This participant talked about shopping for both at the same time:

If I cannot get better after one or two days, then I will switch to another [biomedical] doctor. If that doctor fails too then I will switch to another one. At the same time I will also see a Chinese medicine practitioner as well. Taking western [biomedical] medication hurts my body; it makes my body weaker, especially if I take medications from different doctors at the same time. Chinese medicine is different; it can strengthen your body. This will definitely help me to fight against diseases. Also, when western medicine fails, Chinese medicine can help. Therefore I always see a Chinese medicine practitioner when I am seeing western [biomedical] doctors. Just like my bladder – I went to Chinese medicine practitioner as well as seeing western doctors. Chinese medicine can be a good back-up for western medicine in my case, since western medicine still fails to treat my bladder problem. [P6]

Although the participants always doctor shopped when seeking biomedical treatment, they rarely did the same for TCM treatment. One participant explained the difference:

Western [biomedical] doctors receive the same training and education in universities. They graduate with the same qualification. Therefore, it does not really matter which doctor you see, because what you can expect from their treatment is more or less the same. Chinese medicine is different. The training and education can vary greatly. Some Chinese medicine practitioners receive their education in universities, but others receive their training from their fathers or from some unregistered Chinese medicine schools. Also, unlike western medicine, Chinese medicine practitioners do not have any scientific appliances and tests to help them in the diagnosis. All the diagnoses require their senses, experiences, skills, and abilities. Therefore, I do not mind trying different western doctors, because there will not be much harm even if I see a bad one; but I will stick to one Chinese medicine practitioner if he or she is a good match with me, because I never know if I can get a better and more-qualified one if I try a new one. [P11]

## Discussion

Although the participants were suffering from chronic bladder dysfunction, which in theory requires continuous care from a single healthcare provider [18], doctor shopping behavior was very prevalent. This behavior was influenced by five intertwined themes. Besides the perception that participants did not feel the need of having a regular primary care doctor, their situated social environment, illness experiences, personal cultural preference, and cultural beliefs also combined to generate their doctor shopping behavior.

Participants’ lack of perceived need for a regular primary care provider was a prevalent factor behind doctor shopping behavior. All of them were under long-term treatment plans at public hospitals, and therefore they did not perceive any need for a regular primary care provider to take care of their bladder condition. This is consistent with previous study findings that chronically ill patients often prefer to receive follow-up treatment from specialist outpatient clinics, because of their higher confidence in the capability of public hospital care [9,29].

Many participants did have regular doctors, but these were specialists consulted to treat more serious health problems. In contrast, participants thought that primary care doctors were only there to treat "simple" illnesses, which they perceived not to have significant impact on their health. Hence they were not fastidious in selecting a primary care doctor, and rarely considered establishing a long-term therapeutic relationship. As a result, doctor shopping in primary care was popular. This echoes the findings of previous literature, showing that patients are more likely to doctor shop for treatment of less serious diseases [29].

Surprisingly, past research shows that there is actually high satisfaction with primary care doctors in Hong Kong, and that the perceived prevalence of doctor shopping is actually a mistaken myth among Hong Kong people [30]. However, this finding fails to apply on the participants. Because of their unpleasant experience with primary care doctors in treating their bladder condition, lack of confidence in primary care doctors was strong among the participants, which reinforced their doctor shopping behavior, as well as their tendency to only seek treatment from primary care physicians for "simple" diseases. Instead, other environmental factors such as the convenience of a clinic’s location and consultation times often played a deciding role when shopping for and choosing a primary care doctor [31].

Another crucial environmental factor was work-provided medical insurance. Although a minority of participants did have a regular primary care doctor, they were forced to switch to other designated doctors under their employers’ medical insurance schemes. Participants had to adapt to the style of the new doctors, with whom they often found difficulty "matching" in terms of medication, style, and service. These difficulties further encouraged them to keep shopping for a match doctor. It also meant participants might have to switch to another doctor if they moved to a new job. As a result, the restricting terms of their medical benefits could impede the participants’ ability to establish long-term relationships with their primary care doctors. This finding is contrary to previous studies, which indicate that people with work-provided medical insurance have a greater tendency to consult the same doctor [29]. Work-provided medical insurance schemes that allow employees to select their own doctors for treatment might serve to decrease doctor shopping behavior.

As some participants indicated, seeking treatment was similar to having a date or even a speed dating with a doctor. Searching for a good match was a notable personal cultural preference and cultural belief among the participants, which influenced their doctor selection. Similar to previous studies [29], the participants defined "match" as treatment efficacy as well as a doctor’s friendly and sincere attitude. If either of these elements failed to fit participants’ needs and expectations it could motivate their doctor shopping behavior. Previous literature confirms that large gaps in communication between doctors and patients [2] and patients’ inability to understand doctors’ treatment [10] are both significant in generating doctor shopping behavior. Negative interactions with doctors can also induce patients to doctor shop [32]. This is consistent with my study’s findings: participants were driven to doctor shopping when physicians failed to understand their bladder symptoms, or when they were shamed or made to feel embarrassed by their doctors. Besides receiving efficacious treatment, patients often expect good personal communication with their doctors [31], even if that communication is not necessarily related to the treatment itself [33]. Being able to establish a good relationship with their doctors, or even friendship, is perceived as an important element within a satisfactory treatment process [34]. This can enhance patients’ positive feelings, and very often the treatment efficacy and outcome [34], which supports the tradition of having family doctors.

The concept of a good patient-doctor match is particularly emphasized among patients in Chinese communities, due to the cultural value placed upon human relationships. "Match" is one of the crucial elements of human connection in Chinese societies. Since the family doctor system is not yet well established in most Chinese communities, Chinese patients often use human relationships to determine a good match doctor. Failure to find this match can prompt people to keep shopping for a more suitable doctor.

The participants’ unpleasant treatment experiences negatively influenced their sense of match with primary care doctors. The hierarchy and power differentials between doctors and patients intensified their unpleasant experiences, with doctors’ higher social status placing them in the dominant position during the treatment process. As mentioned before, the failure of many primary care doctors to understand the participants’ suffering or to communicate with them effectively served as a remarkable barrier in establishing long-term therapeutic relationships. These experiences were perceived as mismatch, which contributed to participants’ doctor shopping behavior. In contrast, patient satisfaction significantly discourages doctor shopping behavior [35]. Unfortunately, miscommunication between patients and doctors is common [22,23]; this is particularly true for OAB patients [24], and patients with OAB are commonly dissatisfied with the care they receive [23].

The unpleasant treatment experiences that participants faced also arose from insufficient knowledge about OAB among primary care doctors. Patients with OAB are often under-diagnosed in primary care settings, and doctors’ approach to OAB patients is shown to contribute to that under-diagnosis [36]. In addition to the common perception that primary care doctors lack competence, the under-diagnosis reinforced participants’ feelings that they were not being provided effective treatment, and thus had not found a good match doctor. Because bladder dysfunction is highly prevalent among patients at primary care clinics [37], enhancing primary care doctors’ knowledge of OAB can prevent patients from leaving undiagnosed and untreated, and reduce doctor shopping behavior.

Due to their cultural beliefs about medicine, participants often switched between biomedicine and TCM for a single disease episode. The participants commonly perceived biomedicine as "harmful" to the body, and therefore sought TCM as a follow-up or concurrent treatment. Complementary and alternative medicine (CAM) is often used when biomedical treatments fail [38]. Since TCM is one of the most popular forms of CAM in Hong Kong [39], and because of the unpleasant treatment experiences in biomedicine for their bladder condition, participants also shopped for TCM alternative.

Confidence in the medical system played a significant role in participants’ doctor shopping behavior. Past studies note that people who doctor shop tend to have less positive attitudes toward the medical system [12]; however, participants in this study practiced doctor shopping because they did have confidence in the qualification of biomedical doctors. Because all biomedical doctors are trained at university medical schools with standardized examinations, qualifications, and registration system, participants had a high degree of confidence in biomedical doctors and were comfortable switching between them. They were not aware about the possible negative impacts of doctor shopping. On the other hand, participants perceived TCM as less standardized. The lack of confidence in TCM training meant participants dared not switch TCM practitioners lightly, and made them stick to their own regular TCM practitioner.

Doctor shopping behavior can cause potential risks to a person’s health. It is of particular note for the participants since they were on treatment for their bladder condition, but concurrent biomedical and TCM treatments were not uncommon among them; in some cases, participants sought concurrent treatment from different biomedical doctors. Without notifying their treatment providers, this could lead to possible adverse drug interactions [6]. A family doctor model is therefore recommended to reduce such risks [6]. Alternatively, more education is needed to inform patients about the importance of discussing their medications with new treatment providers, particularly in societies where doctor shopping is commonly practiced.

### Limitations

The findings of this article were based on a sample of 30 patients with OAB from a patient self-help group in Hong Kong. Also, all the participants came from a single study site, and patients outside of the self-help group were excluded from sampling. Further research on a larger sample of patients involving different study sites may add more credibility to the study of doctor shopping behavior in different therapeutic settings. On the other hand, the whole research including data collection and data analysis was conducted by single researcher, who was the interviewer and the writer of this article. Although this may impose bias and subjectivity in the data collection and analysis, recoding of interviews were performed to overcome this potential limitation. Also, having a single researcher to conduct the whole research can ensure consistency and quality of interviews and data.

## Conclusions

Although having a regular doctor is recommended particularly for chronically ill patients, doctor shopping behavior is common in Hong Kong. However, the reasons behind doctor shopping are never simple. In addition to the perceptual factors, participants’ social environment, illness experiences, personal cultural preference, and cultural beliefs also contribute to doctor shopping behavior. Due to the participants’ low perceived need for having a regular primary care doctor, other environmental factors such as time, locational convenience, and work-provided medical insurance benefits became decisive for the participants’ doctor shopping behavior. Their unpleasant illness experiences, which were often due to the prevalent lack of understanding about OAB among many primary care doctors, contributed to participants’ personal sense of mismatch with these doctors, which motivated them to shop for others. Patients also ran the risk of potential negative drug interactions by receiving concurrent treatments from different medical care providers.

Overactive bladder is a chronic bladder condition with very limited treatment outcome. Although patients with this bladder dysfunction often require long-term specialty urology treatment, it is still beneficial for the patients to receive continuous, coordinated, comprehensive, and patient-centered support from one regular primary care doctor. As primary care doctors are the first contact of patient care, it is important for primary care doctors to approach overactive bladder patients with understanding and empathy to reduce the motivations of doctor shopping behavior among this population.

## Competing interests

The author declares to have no competing interests.

## Pre-publication history

The pre-publication history for this paper can be accessed here:

http://www.biomedcentral.com/1471-2296/15/27/prepub
